# Retraction: Targeted delivery of 5-fluorouracil, miR-532-3p, and si-KRAS to the colorectal tumor using layer-by-layer liposomes

**DOI:** 10.3389/fbioe.2024.1478182

**Published:** 2024-08-22

**Authors:** Frontiers Editorial Office

The journal retracts the 17 October 2022 article cited above.

Following publication, concerns were raised regarding the integrity of the images in the published figures and the validity of the data in the article.

Following provision of raw data by the authors, the Chief Editor concluded that the article’s conclusions and assertions were not sufficiently supported by the findings from the material provided; therefore, the article has been retracted. The authors did not agree to this retraction.

